# Behind the stethoscope: The hidden struggles and strengths of veterinarians in Italy

**DOI:** 10.1002/vro2.70036

**Published:** 2026-05-07

**Authors:** Selena Russo, Alexia Del Greco, Marco Bani, Federico Zorzi, Stefano Ardenghi, Giulia Rampoldi, Simone Scoccianti, Paolo Galli, Maria Grazia Strepparava

**Affiliations:** ^1^ School of Medicine and Surgery University of Milano—Bicocca Monza Italy; ^2^ Department of Earth and Environmental Sciences University of Milano—Bicocca Milano Italy; ^3^ Department of Pedagogy Psychology and Philosophy University of Cagliari Cagliari Italy; ^4^ Clinical Unit ‘Psicologia Clinica’ Fondazione IRCCS San Gerardo dei Tintori Monza Italy; ^5^ Pediatric Hematology Unit Fondazione IRCCS San Gerardo dei Tintori Monza Italy; ^6^ Private Practice Florence Italy; ^7^ Marine Research and Higher Education Center Magoodhoo Island Faafu Atoll Maldives

**Keywords:** clinical psychology, qualitative exploration, Italian veterinarians, veterinarians' wellbeing

## Abstract

**Background:**

Veterinarians play a crucial role in public health, animal welfare and human–animal relationships, yet their work involves increasing emotional, structural and ethical challenges. This study explored the lived experiences, perceptions, and needs of veterinary professionals in Italy to understand how these dimensions shape wellbeing and professional identity.

**Methods:**

A qualitative cross‐sectional design was adopted. Twenty veterinarians were recruited through purposive and snowball sampling. Semi‐structured online interviews were audio‐recorded, transcribed verbatim and analysed thematically using the framework method.

**Results:**

Four interconnected themes emerged: (1) structural vulnerabilities, including workload, economic pressure and contractual insecurity; (2) emotional labour and moral distress, particularly around euthanasia, client grief and ethical decision making; (3) interpersonal complexity, involving emotionally charged client relationships and team dynamics; and (4) professional identity and societal (mis)recognition, reflecting frustration with misconceptions and lack of institutional support. Communication emerged as a transversal challenge, with many participants reporting insufficient training for emotionally demanding interactions.

**Conclusions:**

Veterinary professionals face multifaceted challenges that affect their emotional health, job satisfaction and social recognition. Findings underscore the need for improved education, organisational restructuring and stronger institutional commitment to wellbeing. Enhancing public understanding of the veterinary role and promoting healthier human–animal relationships may support a more sustainable professional context.

**Limitations:**

Despite diverse participant representation, the small, self‐selected sample and predominance of female participants may have influenced findings. While not statistically generalisable, the study offers transferable insights into veterinarians’ lived experiences and points towards future comparative research across contexts.

## INTRODUCTION

Human‒animal relationships are undergoing a significant cultural transformation on a global scale. In recent years, the presence of animals in people's lives has become increasingly meaningful, not only as companions but as fully integrated members of family systems and social spaces.^1^ This trend is reflected in the growing number of pet owners, the diversity of animals kept as companions, and the increasing investments in their care and wellbeing. In a report^2^ by Health for Animals of 2022, data show that over half of the world's population shares their homes with pets. In just the United States, EU and China, families care for more than 500 million dogs and cats. In the United States alone, 70% of households had at least one pet in 2021. For what concerns Italy, people owned over 10 million cats and roughly nine million dogs in 2023. Owning exotic or non‐conventional pet animals represents an increasing phenomenon, with Italians living with approximately 30 million fish and 13 million birds.^3^ Interestingly, eight out of 10 pet owners in Italy report to consider their pet as a family member,^4^ and such importance and attention are well reflected in the amount of money they spend on them. In 2022, over 2.7 billion euros were spent on dog and cat food products only in Italy.^5^

The emergence of multispecies families exemplifies this transformation, as animals increasingly participate in household routines, emotional landscapes and spatial design.^6^ Urban environments are progressively adapting to this change, developing pet‐friendly infrastructures, from parks and cafés to travel services and hospitality venues, designed to accommodate both humans and their non‐human animal companions.^6^

Importantly, this cultural shift is not limited to companion animals. In Western societies, increasing attention is being directed towards the welfare, rights and sentience of animals more broadly, including those raised for food, used in entertainment or scientific research, or living in wild or farmed conditions.^7^, ^8^ Ethical debates, public campaigns and institutional changes reflect a growing awareness of animals as sentient beings whose treatment deserves scrutiny, regardless of their social categorisation or economic role.^9^, ^10^, ^11^

This reconfiguration of human‒animal relationships calls for a deeper understanding of the actors most directly involved in animal care and advocacy, among whom veterinarians occupy a central and complex position.

Veterinarians indeed play a pivotal role in mediating the relationships between humans and animals. Their responsibilities extend beyond clinical care, encompassing public health, food safety, environmental protection and scientific research.^12^, ^13^ Across Europe, there are more than 328,494 veterinarians.^14^ As shown in a Federation of Veterinarians in Europe recent survey^14^ (2023), most veterinarians work in clinical practice with small companion animals (63%), followed by those working in public service (14%), education and research (6%) and industry (4%). Despite their essential role, veterinarians often face significant professional and emotional challenges. The nature of their work includes high emotional demands, long hours, financial pressures, exposure to animal suffering and ethically complex interactions with pet and animal owners.^15^, ^16^, ^17^ These conditions contribute to elevated risks of stress, burnout, compassion fatigue and moral distress, and have raised increasing concern among scholars and institutions regarding veterinary wellbeing and long‐term career viability.^18^ While similar trends have been observed across various countries, it is also important to consider the unique cultural and structural features of the veterinary profession in Italy.

In Italy, the veterinary profession is shaped by the country's public health system, the *Servizio Sanitario Nazionale*, which positions veterinarians not only as private practitioners but also as public officials embedded within the *Servizi Veterinari* of the Local Health Authorities. According to recent data,^19^ there are approximately 35,000 registered veterinarians in Italy, the majority (about 82%) working in self‐employed private practice,^20^ with the remainder employed in public health, academia, armed forces or industry. This systemic arrangement creates distinct professional challenges. Public‐sector veterinarians face heavy administrative and inspection workloads, with high bureaucratic pressure and ethical responsibility, while private practitioners are financially dependent on clients and therefore confronted with dilemmas such as owners having to afford treatment.^21^ While a substantial portion of the Italian population (approximately 35%) recognises veterinarians’ work as useful or professional, relatively few appreciate and recognise its complexity or find it fascinating.^22^ At the same time, even though the public places high importance on activities that veterinarians perform, such as quality control in food production, protection of endangered species, and the health of companion animals, there is often limited awareness that these critical tasks fall within the veterinarians’ remit.^22^ This gap between the visible societal value of their work and the public's understanding of their professional responsibilities may contribute to the undervaluation of veterinarians in society.

Growing international concern for the mental health and long‐term career viability of veterinary professionals has led to several recent studies.^15^, ^16^, ^17^, ^18^ However, little is known about how veterinarians in specific cultural and institutional settings experience and interpret these pressures. The Italian context, with its mixed public–private structure and unique social representations of veterinary work, remains underexplored. Understanding how Italian veterinarians construct meaning around their roles and manage the emotional, relational and organisational aspects of their work can provide deeper insights into the profession's evolving identity and needs.

This qualitative study aims to gain a deep understanding of the experiences, beliefs and challenges of veterinary practitioners in Italy, focusing on how they navigate the emotional, educational and policy‐related dimensions of their work. Through semi‐structured interviews, the present study seeks to amplify the voices of those who care for animals and to offer insights that may inform supportive educational, institutional and policy actions at the national and international levels.

## MATERIALS AND METHODS

### Study design and setting

This study adopted a qualitative, using in‐depth semi‐structured interviews and followed the best practices in qualitative research and COREQ recommendations. Before the interviews, all the participants were informed about the study's aims and provided informed consent digitally. Ethical approval was granted by the Ethics Committee of the University of Milano‐Bicocca (protocol no. 0173021, 14/12/2022).

### Sample and recruitment

A purposive sampling strategy was used to recruit participants. The inclusion criteria required participants to be registered veterinarians living and working in Italy, while the exclusion criteria included retired veterinarians or those practicing abroad. No inclusion criteria were imposed based on age, years of professional experience, specialisation or geographic location.

Approximately 35 veterinarians were identified through online searches, social media and snowball sampling techniques and were contacted via email, telephone or text message. To ensure variation in professional backgrounds, 35 veterinarians were intentionally selected to reflect the diversity of the profession (e.g., companion animal practice, exotic animal practice, public health, research and academia). Of these, 20 agreed to participate (response rate: 57%). A sample of 20 participants was considered appropriate given the structure of the interview guide and the scope of the study. Data saturation was considered achieved when no new codes or meaningful conceptual insights emerged during the final interviews. This point was reached after approximately 18 interviews and confirmed during the final two. This approach is consistent with established literature on saturation in qualitative research^23^, ^24^ and aligns with existing literature indicating that 20‒50 interviews are generally sufficient for qualitative research using semi‐structured interviews, depending on the level of structure and thematic focus.^25^, ^26^ Interviews were conducted between May 2023 and October 2024.

### Data collection

The interviews were conducted via Google Meet or Microsoft Teams, ensuring flexibility and accessibility for participants. Interviews lasted between 40 and 60 minutes and were conducted in Italian by S.R. and A.D.G., both psychologists, using a semi‐structured format, guided by a topic guide (Table 1) developed collaboratively by the research team, including psychologists, a veterinarian and an ecologist. The development of the interview guide was informed by existing international literature on veterinary wellbeing and theoretical considerations^15^, ^16^, ^17^, ^18^ and drawing on the veterinarian's clinical experience, the ecologist's direct work with veterinarians, and the psychologists’ expertise in counselling and supporting veterinary staff. The guide explored five main thematic areas: professional experiences and identity, beliefs and attitudes towards human‒animal interactions, public education on animal welfare, training and professional development on communication and education aspects, and perspectives on relevant policies.

**TABLE 1:** Thematic guide.

Topic	Examples of questions and prompts used
Experiences working with animals: Professional definition and mission Rewarding aspects Challenges, unmet needs, concerns	‐ Could you tell me more about your professional life and your daily tasks?—What are the rewarding aspects of your profession, if any? ‐ What are the main challenges, if any, you encounter or have encountered in your profession when working with animals? ‐ What, in your view, would help mitigate these challenges? ‐ What would you like to see happen that, in your opinion, would make your work easier or help you fully accomplish your mission/task?
Beliefs and attitudes about human‒animal interactions: Characteristics of functional and healthy human‒animal interaction Connectedness to animals Perception of animals’ role in our society	‐ In your opinion, what makes a human–animal interaction functional and positive? And why? ‐ Do you think human animals can form meaningful bonds with non‑human animals? Why (not)? ‐ Is the possibility of connecting and bonding with non‑human animals related to the animals’ characteristics? If yes, which ones? ‐ How connected do you feel to animals? ‐ (If the participant has not touched on this earlier) How do you think people in our society are generally connected to animals?
Educating the public: Perceived importance of educating the public about animal welfare and needs Perceived responsibility of self or other professionals Perceived most appropriate audience to address experiences in educating the public and the biggest challenges faced Unmet needs, perceived skills and self‐efficacy	‐ Do you think it is important to inform and educate the public about issues related to animal welfare? Why (not)? ‐ Who, in your opinion, has or should have this responsibility? ‐ In your view, who would be the most appropriate audience to address if the goal is to promote positive human–animal interactions? Why? ‐ Do you think it is important for people who work with animals, like you, to play a role in educating the community? Why (not)? ‐ Have you had any experience in disseminating information or educating the general public? If the participant has previous experience: ‐ Could you tell me more about these experiences? ‐ What were the main challenges or problematic aspects you encountered, if any? ‐ What would have helped you in that situation? If the participant has not had previous experience, ‐ Would you like to engage in public outreach or play a role in educating the public about your activities or field of expertise? ‐ Do you feel prepared and qualified to communicate with the general public or to take on an educational role regarding your activities/field of expertise? ‐ Why (not)? ‐ What would make you feel more comfortable or well‑prepared for this task?
Training on communication and educating: perceived needs and preferences: Training received Training desired Best practices and modalities to receive training	‐ What kind of training have you received in terms of communicating to your audiences? ‐ Do you feel you need—or would have benefited from‐ training on how to communicate information to non‑experts or to a specific audience? If yes: ‐ Which aspects or topics would you like to receive training on? ‐ In your opinion, which delivery format would be most appropriate and support effective training?
Policies: Perceived role and knowledge of policy makers when legislating on animal welfare and functional human‒animal interactions	‐ What do you think authorities should know when they legislate on animal welfare and nature conservation strategies? ‐ Which aspects, currently not considered, should authorities take into account when legislating on animal welfare and nature conservation strategies? ‐ In your opinion, what is missing at the moment?

Before formal data collection, the interview guide was pilot tested with two veterinary professionals to ensure clarity, relevance and flow. Minor refinements were made based on their feedback, particularly in the wording and sequencing of questions.

While the interviewers were not veterinarians, their backgrounds in human healthcare shaped their sensitivity to emotional and relational dynamics. However, this positioning also brought potential biases, including the assumption that veterinarians’ emotional burden mirrors that of other health professionals or a tendency to prioritise psychological over systemic aspects of the profession. To mitigate these risks, the authors engaged in regular reflexive discussions throughout the research process, critically examining how their assumptions, disciplinary lenses and interview style might influence the data. This reflexive stance helped ensure that the analysis remained grounded in participants’ lived experiences and language.

All sessions were audio‐recorded and transcribed verbatim using Trint—Automatic transcription software. Transcripts were anonymised and reviewed for accuracy. No member of the team had an existing relationship with any of the participants.

### Data analysis

The interview transcripts were analysed using the framework analysis approach,^27^ which is well suited for applied qualitative research involving pre‐defined topics while allowing new themes to emerge inductively. Data analysis therefore combined inductive and deductive strategies. Two researchers (S.R. and A.D.G.) independently coded the transcripts after an initial familiarisation phase with the data by double reading all 20 interviews and producing initial notes. Similar codes were grouped into abstract categories, which were iteratively refined into sub‐themes and overarching themes through discussion and comparison. The process involved multiple rounds of discussion within the research team to ensure consistency and depth of interpretation. Furthermore, a third researcher (M.B.) reviewed the coding to ensure consistency and inter‐coder reliability across the dataset.

The transcripts of the interviews were reviewed exclusively by the research team. The participants were not asked to review their transcripts.

The analysis framework and final interpretations were reviewed and approved by all co‐authors. Transcriptions were prepared and analysed in Italian, and quotes were translated into English during manuscript preparation with the support of a bilingual English‒Italian speaker as a back translator for validating our translations.

## RESULTS

The participants' main characteristics are reported in Table 2. Analysis revealed four interrelated themes: structural vulnerabilities in veterinary practice; emotional labour and moral distress; navigating interpersonal complexity; and professional identity and societal (mis)recognition.

**TABLE 2:** Participants’ characteristics.

Sample	Total, *n* = 20
Age (years)	Mean age: 40 (SD = 8.8)
Sex	Females = 15 Males = 5
Workplace (some participants have more than one)	University = 4 Clinics (small companion animals) = 16 Public health = 2
Geographical area	Northern Italy = 14 Centre = 1 Southern Italy = 5
Public versus private practice	Public health = 3 Private practice = 17
Specialty (some participants hold multiple specialisations)	Neurology = 2 Ecography = 3 Infectious diseases = 1 General internal medicine = 6 Cardiology = 4 Dermatology = 2 Reproduction = 2 Pathological anatomy oncology = 1 Anaesthesiology = 1 Management = 1

We developed a coding tree to document and visually represent how raw data were organised into themes. This coding tree included three hierarchical levels: codes, sub‐themes and themes. A summary of this structure is available in the Supporting Information S1.

### Structural vulnerabilities in veterinary practice

Veterinarians consistently described their work environments as demanding and unstructured, with ‘unexpected emergencies’ and ‘after‐hours calls from clients’ preventing any real work‒life balance. The absence of clear boundaries and a high volume of overlapping requests were seen as major stressors. One participant noted, ‘Clients call you at any time and you never take a break from work’ (Participant 14). The daily routine was frequently described as chaotic, with professionals being ‘expected to handle multiple patients at once’ and constantly shifting between tasks. As another described, ‘You're in your office doing your work, and a colleague walks in with a request, another one arrives, and then you get a call from reception. It's all very demanding’ (Participant 15).

Workload and time pressure were compounded by the perception that clients expected immediate and exhaustive care. As one interviewee reported, ‘Sometimes I see patients in two rooms at the same time. While I'm talking to one client, I'm already thinking about the examination I need to do on another dog’ (Participant 18). This sense of fragmentation and overload often led to emotional fatigue and frustration. Young veterinarians often reported a mismatch between responsibilities and preparedness, particularly in emergency settings. As one put it, ‘I am doing night shifts in emergency care where I am completely alone, entirely on my own, and I don't have training in emergency care’ (Participant 6). Their requests for support were ‘deliberately ignored’ due to the need to cover essential shifts, leaving them feeling vulnerable and professionally exposed.

Financial pressures also emerged as a central concern. Rising operational costs and low compensation rates made many feel that their profession was economically unsustainable. ‘The costs of practising the profession have become truly exorbitant compared to how much actually comes into the till’ (Participant 4), one clinician lamented. Participant 9 reported, ‘Today, some young graduates are simply not willing to put up with this (situation) anymore, so they choose other careers. We hear from many veterinary graduates who'd rather not survive on €50 a night with the full responsibility of a night shift and a completely disrupted life. After all, for the same €50, they'd rather work as a supermarket cashier. It's not just about being underpaid, it's about being undervalued’. Others highlighted the dissonance between their level of responsibility and income, especially compared to other sectors, ‘You're responsible for life and death, and still, specialised workers outside medicine earn more than us’ (Participant 20).

Contractual instability further compounded these difficulties. Many participants reported being treated like employees while bearing the fiscal burdens of freelancers. ‘You don't have the benefits of being an employee, but you have all the disadvantages of being self‐employed’ (Participant 13), one veterinarian explained. The lack of autonomy, combined with obligations such as fixed hours and non‐compete clauses, contributed to a widespread sense of professional entrapment. Financial and contractual constraints may lead female veterinary practitioners to not disclose their pregnancies, as reported by two participants.

Not all the participants reported the same level of structural burden. Those occupying managerial or ownership roles (Participants 12 and 19), as well as veterinarians with a strong public or media presence (Participant 11), described greater autonomy and fewer financial or time‐management pressures. Their accounts suggest that positional authority and visibility may buffer certain systemic stressors commonly experienced by others in the field.

### Emotional labour and moral distress

In addition to structural stressors, veterinarians described a profound emotional burden as a core aspect of their professional experience. The dual responsibility of caring for both animal patients and their human companions emerged as a significant source of strain. As one participant explained, ‘You don't just deal with the illness of the animal you're treating, but you also come into contact with an entire family, with the personal situation of the owners’ (Participant 7).

Participants recounted frequent exposure to emotionally charged situations, particularly in end‐of‐life care, which were described as mentally exhausting and deeply affecting. A young professional shared, ‘Mentally, euthanising a patient is heavy. And you don't get used to it. The truth is, you don't get used to it’ (Participant 6). Several interviewees reported feeling unprepared for such moments, having received no formal training on supporting grieving clients or managing their own emotional responses, ‘University doesn't prepare you for this interaction. You study diseases, but then seeing the animal and having to put it to sleep … it's a hard impact, especially at the beginning’ (Participant 1).

Participants also described intense emotional entanglement in emergency scenarios. One veterinarian recalled a distressing episode during a failed resuscitation attempt, ‘I had to calm her down, give her water, and I hugged her because she was very, very shaken … I just wanted to sleep because I felt so empty from the emotional intensity of the event’ (Participant 6).

Ethical dilemmas further heightened the emotional toll. Many professionals reported experiencing moral injury, especially in cases involving client non‐compliance or economic constraints that limited treatment options. As one participant put it, ‘Sometimes, we are almost faced with a kind of blackmail: “No, I can't pay. So, will you let my animal die because of money?”’ (Participant 7). Others expressed distress over excessive anthropomorphism or pressure to perform unnecessary procedures. On the one hand, ‘The animal is seen more like a child, which makes it difficult to interact with the owner and, well, to communicate bad news that is not easy to face for them. So this part is the most difficult and stressful, utterly exhausting’ (Participant 1); on the other hand, ‘Very often, some owners seem to believe that requesting euthanasia is as simple as ordering a pizza’ (Participant 13). Interestingly, Participant 18, who works in veterinary oncology, described their emotionally intense field as a source of deep professional fulfilment. Despite the frequent exposure to loss, they referred to their practice as a ‘happy oasis’ due to the profound trust and relational depth shared with pet owners. As a successful trust‐building strategy, the veterinarian described dedicating as much time as necessary to their consultations, allowing space for emotional connection and reflective engagement with both the animal and the owner. This trust is further strengthened by honesty, shared awareness of prognosis, and the owners’ willingness to emotionally and financially engage with the care process, ‘They put themselves in your hands and say, “I'm entrusting you with one of the most precious things I have” […]. Even though things often end badly—it feels different’ (Participant 17). This account contrasts with narratives of emotional exhaustion, suggesting that meaningful, undisturbed time with clients can serve as a protective factor against moral distress.

A lack of psychological training and support tools often exacerbates these emotionally demanding situations. Several participants highlighted the absence of university preparation or institutional guidance in managing either their own emotional states or those of their clients. One commented, ‘There's little talk about psychological approach and the psychological management of emotions … you're at the mercy of anyone who might find solutions’ (Participant 17). Another reflected, ‘We don't have the tools to face a patient who is screaming or desperate. I freeze. I take the insults because I don't know what else to do’ (Participant 18). Participant 6 well summarised their perceived lack of preparation and tools to manage communication and relational aspects of the job, ‘They don't teach you this anywhere—meaning there aren't any university courses that specifically teach you how to manage not only your own emotions when facing work‐related difficulties, whether it's performing euthanasia or handling conflicts, but also how to deal with the emotional side of interacting with clients’. Participants 6 elaborated further ‘… Even things like team building, how to work in a group, how to manage conflicts. In a clinic where none of this is provided, you're left to rely on your own personality, character, past experiences and abilities’.

Throughout the interviews, the emotional and moral demands of veterinary work were not only described as intense and enduring, but also largely invisible and seldom acknowledged or supported by institutions and frequently misunderstood by the public. Emotional labour, although central to the veterinary role, often remains unspoken and unsupported.

### Navigating interpersonal complexity

Veterinarians reported that managing relationships with clients, colleagues and within teams constituted one of the most complex and demanding aspects of their professional lives. These interpersonal dynamics were described as both rewarding and draining, with trust, communication and expectations playing key roles in shaping daily interactions.

Relationships with clients were frequently described as a double‐edged sword. Many participants found satisfaction in long‐term connections based on trust and shared care. As one interviewee expressed, ‘It's wonderful to have owners and patients you can follow and see improving … Even accompanying owners and patients during the final moment of euthanasia, while painful, can be beautiful when they say it's something they remember with fondness’ (Participant 14). Others found joy in guiding families, particularly children, in caring for their pets, describing it as ‘satisfying to help owners understand their animals as sentient beings that need attention’ (Participant 6).

However, these relationships were also described as emotionally and professionally challenging, with participants speaking of clients who distrusted veterinary advice, questioned costs or resisted treatment plans. One participant explained, ‘You try to explain a serious condition and they reply with something they read online. I've had to say, “Well yes, how stupid I was to waste my life studying, when Google could treat your dog”’ (Participant 18). Client expectations often seemed unrealistic and conflicted, ‘They don't want to spend money, but they want the best possible service. So, I pay and you have to be at my feet, my slave—you have to respond whenever I want’ (Participant 13). These misunderstandings could result in communication breakdowns and emotional strain, as one clinician shared, ‘We are constantly insulted—insults are a daily occurrence. I don't know what to say or do, so my weapon is immobility. I freeze and take it all’ (Participant 18).

The emotional meaning of animals within family systems further complicates these interactions. Veterinarians described encountering clients for whom pets represented deceased loved ones, surrogate children or were seen as disposable. The resulting moral dilemmas were often intense, a veterinarian mentioned, ‘Once in the emergency room, I had to euthanase—I remember it clearly—it was a dog. I had diagnosed a tumour, and then the lady came out saying, “Oh wow, it's the same tumour that its owner had who died 3 months ago”’ (Participant 1). Others expressed frustration when treatment decisions were blocked due to family dynamics, economic limits or denial, ‘Many times a patient could be saved, but this doesn't happen because the owner doesn't cooperate’ (Participant 16), ‘Some people just tell me, “Well, it is what it is, just euthanase it”. And every time I respond, “you're not asking for gas at the petrol station. If I don't want to perform the euthanasia, I won't. It's not your right to demand that I end your animal's life”’ (Participant 4).

Relationships with colleagues also emerged as a source of stress. Several participants described a climate of mistrust, individualism, and competition within clinics. One noted, ‘The prevailing atmosphere is “everyone covering their own backs” … the risk of daily reprimands is very high’ (Participant 6). Another recounted nightmares caused by workplace toxicity, ‘I left after two months because I had nightmares at night … there was no regard for the client, no regard for the animal’ (Participant 20).

Finally, the lack of communication training was identified as a critical gap. Most participants reported never having been taught how to communicate with distressed owners or navigate emotionally intense situations, as Participant 1 stated ‘How to approach the owner—understanding what kind of person you have in front of you—is something you learn on the job. We receive no preparation’. Others emphasised the need for tools to manage difficult conversations, ‘Communicating bad news is the hardest thing for us … no one teaches us, and we don't know how to deal with it’ (Participant 15).

Overall, navigating interpersonal complexity was described as one of the most draining and yet defining aspects of veterinary practice, often described as central to care but often left unsupported and under‐recognised.

### Professional identity and societal (mis)recognition

Veterinarians in this study reflected critically on their professional identity and how it is perceived, and often misunderstood, by society at large. Many described a polarised view of their role. They are either romanticised as selfless animal lovers or seen as service providers whose work should be fast, flawless and affordable. The participants reported that this contradiction undermines both their professional authority and emotional resilience.

Several veterinarians shared their frustration with the public's tendency to equate veterinary work with personal passion, often overlooking the scientific and clinical expertise involved. As one participant noted, ‘As a vet, you're seen as the animals’ friend. Since it's a job of passion, people think they can pay and get everything right away’ (Participant 13). This mindset was perceived as particularly damaging when combined with a transactional attitude, ‘Because I'm paying, I deserve immediate answers, complete availability and flawless outcomes’ (Participant 13). Participants also lamented the lack of public understanding regarding the academic and professional rigour of their training. One veterinarian recalled being asked whether a university degree was required for the job showing how people tend to see the veterinarian less as a doctor, and how that affects everything that comes with it, ‘The other day, a woman asked me if you need to go to university to become a veterinarian (laughs). I mean, she wasn't even particularly old—around 50—so I think people tend to see veterinarians less as doctors (…) So you have to explain to the owner why a certain price is charged, why it's not possible to give simple, clear answers, because unfortunately, animals still can't speak’ (Participant 4).

This lack of recognition extended to misunderstandings about medical uncertainty and complexity, ‘Medicine is not always an exact science. Sometimes you don't reach a diagnosis. But when that happens, owners do not understand. They think you've failed’ (Participant 13). Others described clients demanding immediate, conclusive answers and judging practitioners harshly when outcomes did not match expectations, ‘If you do well, you're amazing. If not, you're terrible, a mess, an incompetent. I'll sue you’ (Participant 18).

There were mixed views on the veterinarian's educational role. While some saw public education on animal welfare as part of their mission, others felt that it should be the responsibility of schools or institutions. Among those committed to educating owners, misinformation was cited as a major challenge, ‘You have to challenge these beliefs passed down like folklore … ideas that have no scientific basis but circulate everywhere’ (Participant 6).

A further source of professional strain came from a perceived lack of cohesion and support within the veterinary community itself. Participants spoke of feeling isolated, disillusioned and unsupported by institutional bodies, ‘We haven't been capable of making people understand the complexity of our work. And there's unfair competition—colleagues lowering prices off the books discredit the profession’ (Participant 4). Another added, ‘Among colleagues there's a lot of conflict. Some work at ridiculously low prices just to get clients. That makes us all look dishonest’ (Participant 17).

Generational tensions within the profession were also prominent. Senior practitioners spoke of having endured years of sacrifice, unpaid labour and long shifts, often with minimal institutional support. Some perceived the younger generation's push for work‐life balance and fair contracts as a lack of commitment. Participant 7, as senior practitioner, explained about their younger colleagues ‘(…) demand more than we did. They're not willing to sacrifice weekends or nights like we had to. This creates a generational gap’. At the same time, several senior participants acknowledged that newly qualified veterinarians are often placed in complex clinical situations without the appropriate preparation or support, ‘You find yourself with recent graduates who aren't ready to manage complex cases but are expected to perform as if they were. Then they get blamed when things go wrong’ (Participant 10). This perspective echoes the frustration younger veterinarians expressed in the interviews, particularly around the mismatch between expectations and preparedness, and the lack of structured mentoring.

These experiences point to a fragmented professional identity, caught between internal divisions, external misconceptions and institutional neglect. While many participants remained deeply committed to their work and its social value, they also expressed a desire for greater recognition, stronger professional unity, and a more realistic cultural understanding of what it means to be a veterinarian today.

## DISCUSSION

This study explored the lived experiences and professional perspectives of a diverse group of veterinarians working in Italy. Through the analysis of semi‐structured interviews, we identified four major, interconnected themes: structural vulnerabilities within the profession; the emotional labour and moral distress involved in veterinary care; the complexity of interpersonal relationships; and tensions surrounding professional identity and societal recognition. These themes offer a nuanced view of the daily realities, challenges and emotional landscapes experienced by veterinary professionals, highlighting a profession deeply affected by institutional pressures and evolving human–animal relationships.

Our findings echo existing international research on occupational stress and wellbeing in veterinary medicine. In particular, they confirm that work‐related stressors such as excessive workload, long and unpredictable hours, low pay and unpaid overtime are consistently reported in the literature.^28^, ^29^ These stressors severely impact work–life balance and wellbeing. Younger and female practitioners, in line with previous studies in both Italy and other countries, appear especially vulnerable to these pressures, reporting greater difficulties in managing work–life integration and emotional regulation.^30^, ^31^

The emotional labour described by participants aligns with Hochschild's theory,^32^ which highlights how workers are required to manage and sometimes suppress their emotions in order to display feelings that align with professional or organisational expectation.

The emotional demands described by participants are also aligned with research on compassion fatigue, secondary traumatic stress and moral injury.^15^, ^16^, ^17^, ^33^ The participants’ accounts also resonate with the conceptualisation of moral injury as outlined in the Moral Injury Events Scale,^34^, ^35^ which distinguishes between moral transgressions committed by oneself, witnessed in others, and experiences of betrayal by trusted authority figures. In this study, veterinarians described witnessing ethically questionable practices—often by senior colleagues or clinic owners—as well as feeling pressured to act against their own moral standards due to organisational or financial constraints. Such experiences exemplify the moral tension embedded in daily veterinary decision making, particularly when financial limitations prevent the delivery of optimal care.

Moral distress often emerged when practitioners were forced to act against their clinical judgement due to owners’ expectations or anthropomorphic attitudes. Such situations can contribute to burnout and mental health concerns because repeatedly compromising one's professional standards generates emotional strain, frustration and a sense of powerlessness. This aligns with prior research showing that veterinarians are at increased risk for burnout and mental health concerns, but often lack adequate support systems.^29^, ^36^, ^37^, ^38^

These challenges are not unique to veterinarians; similar patterns of moral distress, compassion fatigue and burnout have been documented among medical doctors, nurses and other healthcare professionals.^39^, ^40^ However, veterinarians face the additional burden of navigating owner finances and anthropomorphic expectations, which distinguishes their moral labour from that of human healthcare practitioners. Veterinarians may in fact face moral distress when owners insist on anthropomorphic practices that they know conflict with animal welfare.^41^

Interpersonal relationships, particularly with clients, were described as both a stressor and a potential source of fulfilment. While the emotional intensity and complexity of client interactions often led to distress, many participants also identified positive, long‐term relationships as meaningful and rewarding, a duality also highlighted by Connolly et al.^28^ In fact, our participants emphasised that trust, continuity of care, and shared decision making could foster a strong sense of purpose, reinforcing their professional identity and commitment to the field. At the same time, these relationships required substantial emotional labour: practitioners navigated clients’ expectations, anxieties and grief, often absorbing the emotional weight of difficult conversations. This duality (relationships as both supportive and taxing) highlighted the central role of client interactions in shaping veterinarians’ wellbeing and daily professional experience. Social stressors are highly important; our results seem in accordance with the literature that suggests how interactions between people within veterinary settings are the primary source of stress.^42^

Beyond the individual and clinic‐level stressors, our findings must also be situated within systemic structures of the veterinary profession in Italy. In particular, the experience of moral distress and financial strain among veterinarians may be understood in light of the contrast between the human healthcare system, where treatment is largely delivered within public services, and veterinary care, which is entirely financed privately by pet owners. Several participants in fact noted that clients often approach veterinary care with expectations shaped by their experiences in the public health system, where costs are not directly paid by individuals, as services are largely funded through public taxation and national health insurance. Such structural difference means that veterinarians are often compelled to adjust care based on a client's financial capacity, despite their professional judgement, thereby intensifying moral stress and contributing to emotional burden.

The challenges of communication emerged as a recurring issue. Participants frequently reported lacking the tools to manage emotionally intense conversations, communicate medical uncertainty, or address client denial. This is consistent with existing literature emphasising the critical role of communication in promoting client adherence, preventing conflict and reducing legal complaints.^42^, ^43^ Effective communication skills could help clinicians navigate emotionally charged situations, set realistic expectations and de‑escalate tensions; however, when these skills are underdeveloped or unsupported, everyday interactions can easily become a major source of strain. Several participants highlighted how the absence of structured training in communication left them feeling unprepared to manage difficult conversations, contributing to frustration, emotional fatigue and, in some cases, moral distress.

The importance of communication in veterinary care is further intensified by contemporary trends in pet ownership since companion animals are increasingly regarded as family members.^44^ This cultural shift enhances the emotional stakes of veterinary consultations, heightening both the potential rewards of meaningful relationships and the risk of conflict or distress when treatment decisions collide with financial or medical constraints. As in other studies, many participants, especially younger veterinarians, felt unprepared for these demands, pointing to a gap in the veterinary curriculum.^43^

Last, our study sheds light on a broader socio‐cultural tension. The contrast between public representations of veterinarians as altruistic animal lovers and the context of their clinical expertise and professional demands. Participants described a lack of public recognition for the scientific and medical complexity of their work, with owners expecting flawless service at low cost, and often dismissing medical uncertainty as failure. These findings echo Widmar et al.,^45^ who observed that public discourse on veterinarians rarely acknowledges the complexity or challenges of the profession, especially on social media platforms. In fact while veterinarians are often recognised for their role in companion animal care, their equally vital contributions to public health (such as food safety, zoonosis control and animal welfare) remain largely overlooked, reinforcing societal undervaluation and obscuring their broader impact on collective health and long‐term career viability.^45^

### Implications for practice, education and policy

The findings of this study underscore the need to strengthen veterinary education by systematically integrating training on emotional resilience, communication and ethical decision making within undergraduate and postgraduate curricula. The participants highlighted a widespread feeling of being unprepared to manage emotionally intense situations, navigate difficult conversations with clients, or cope with the psychological burden of euthanasia and ethical dilemmas. Addressing these gaps through formal coursework, experiential learning and supervised clinical practice may better equip students for the interpersonal and emotional complexities of veterinary work. Evidence from veterinary education indicates that structured, practice‐based communication training, particularly when incorporating role play, simulation and feedback, enhances students’ communication competencies, confidence and professional readiness.^46^, ^47^ Similar findings from human healthcare education demonstrate that experiential and supervised training significantly improves communication behaviours, empathy and patient‐related outcomes, highlighting the cross‐disciplinary value of these approaches.^48^, ^49^, ^50^ Moreover, continuing professional development programmes should prioritise not only clinical competence but also psychosocial skills, including conflict management, client communication and self‐care strategies. Veterinary schools and professional boards have a shared responsibility to promote a culture of wellbeing, offering access to mental health resources, peer support and spaces for ethical reflection. Institutions should also support early‐career professionals through structured mentoring programmes, as younger veterinarians are particularly vulnerable to emotional fatigue and identity challenges. Finally, the profession may benefit from closer collaboration between veterinary educators, clinicians and policymakers to foster a shared understanding of veterinarians’ roles and to promote public awareness around the complexity and societal value of veterinary work. We also advocate for future research exploring how systemic interventions (e.g., communication training, psychological support and team‐based structures) impact veterinary wellbeing and professional satisfaction.

## STRENGTHS AND LIMITATIONS

This study provides in‐depth insight into the professional lives of Italian veterinarians using a qualitative approach that privileges their lived experiences. The diversity of participants across roles and regions strengthens the transferability of findings. However, the sample size remains relatively small, and participation was voluntary, introducing a possible self‐selection bias. Moreover, the majority of participants identified as female, which may reflect broader trends in the profession but could also influence the type of experiences reported. Comparative studies across different countries and sectors of veterinary medicine could illuminate context‐specific stressors and coping strategies. Additionally, longitudinal designs could help track how professional experiences evolve over time and in response to policy or institutional change.

## CONCLUSIONS

This study provides insight into the complex professional reality experienced by veterinarians, highlighting the emotional, structural, relational and cultural challenges that shape their daily practice. While many participants expressed deep commitment to animal welfare and human–animal relationships, they also reported feeling underprepared for the emotional, ethical and interpersonal dimensions of their work. These findings suggest that veterinary education must evolve to more fully prepare students not only as clinical experts, but also as emotionally resilient professionals, decision makers and communicators. Integrating structured training in communication, psychological skills and ethical reasoning, both at undergraduate and postgraduate levels, may enhance graduates’ readiness to navigate challenges and reduce early‐career vulnerability. In parallel, institutional efforts to promote wellbeing, mentorship and reflective practice are essential to support long‐term professional satisfaction and retention in the field. By situating veterinarians’ lived experiences within these wider systemic and socio‐cultural contexts, our study highlights the need for interventions that operate not only at the individual and clinic levels but also within educational, institutional and policy frameworks.

## AUTHOR CONTRIBUTIONS

*Conceptualisation (lead), methodology (lead), project administration (lead), investigation (lead), formal analysis (lead), writing—original draft (equal) and writing—review and editing (lead)*: Selena Russo. *Conceptualisation (supporting), methodology (supporting), investigation (supporting), formal analysis (supporting), writing—original draft (equal) and writing—review and editing (supporting)*: Alexia Del Greco. *Methodology (supporting), formal analysis (supporting) and writing—review and editing (supporting)*: Marco Bani. *Data curation (supporting) and formal analysis (supporting)*: Federico Zorzi. *Conceptualisation (supporting) and writing—review and editing (supporting)*: Stefano Ardenghi and Giulia Rampoldi. *Resources (supporting), formal analysis (supporting) and writing—review and editing (supporting)*: Simone Scoccianti. *Supervision (supporting), conceptualisation (supporting), methodology (supporting), formal analysis (supporting) and writing—review and editing (supporting)*: Paolo Galli. *Supervision (lead), conceptualisation (supporting), methodology (supporting), formal analysis (supporting) and writing—review and editing (supporting)*: Maria Grazia Strepparava.

## CONFLICTS OF INTEREST

The authors declare they have no conflicts of interest.

## ETHICS STATEMENT

Ethical approval was granted by the Ethics Committee of the University of Milano‐Bicocca (protocol no. 0173021, 14/12/2022).

## Supporting information




**Supporting File 1**: vro270036‐sup‐0001‐SuppMat.png

**Supporting File 2**: vro270036‐sup‐0002‐SuppMat.docx

**Supporting File 3**: vro270036‐sup‐0003‐SuppMat.pdf

## Data Availability

Due to privacy reasons, no additional data are available.
