# Supplementary figures and images for: Behind the stethoscope: The hidden struggles and strengths of veterinarians in Italy
Source: Vet Rec Open. 2026 May 7;13:e70036. doi: 10.1002/vro2.70036 (PMC13150477; doi:10.1002/vro2.70036)

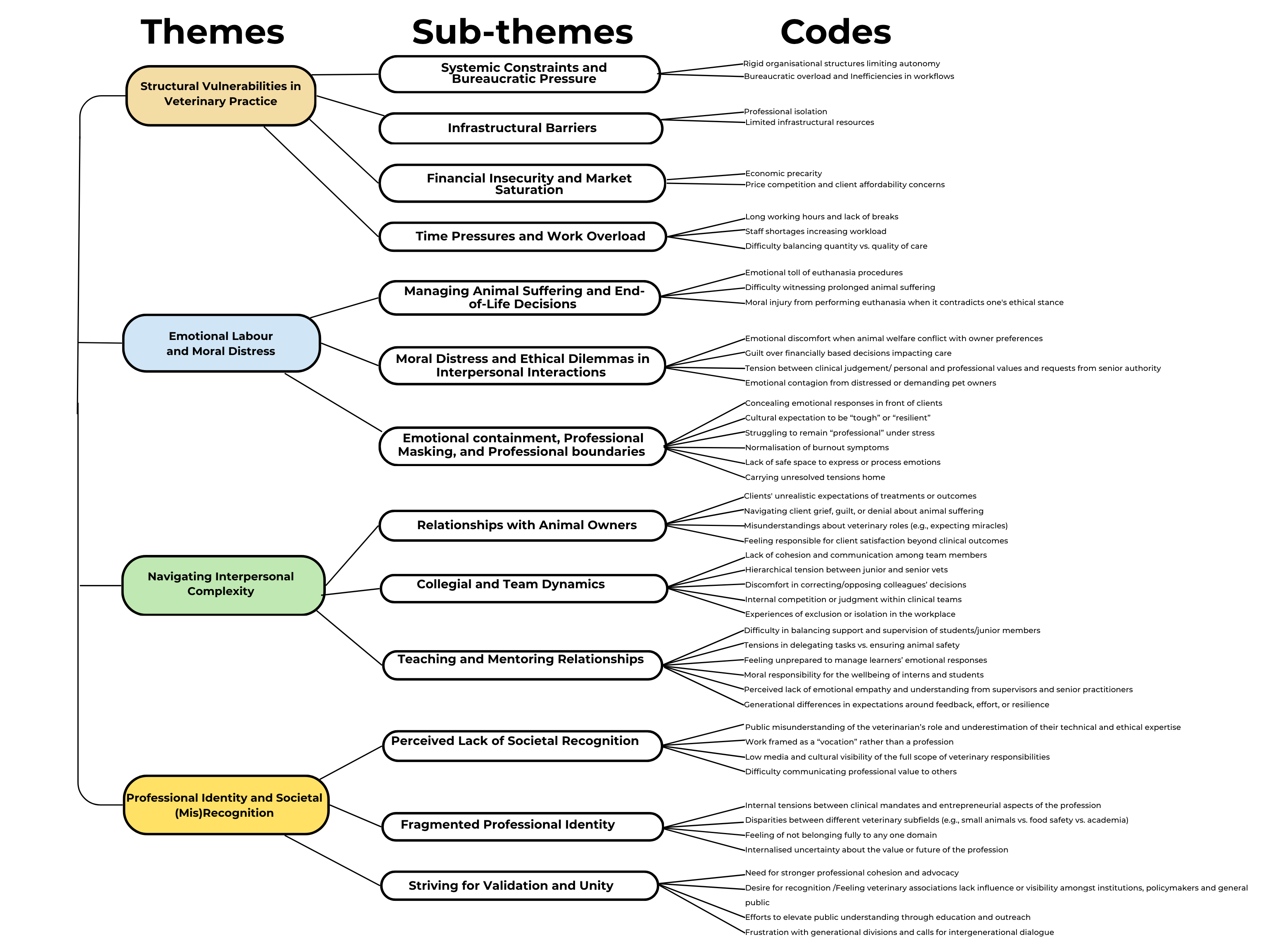

Supplement: Supplementary file 1 — Supporting File 1: vro270036‐sup‐0001‐SuppMat.png [file VRO2-13-e70036-s002.png]

Supplementary File 1. Coding Tree


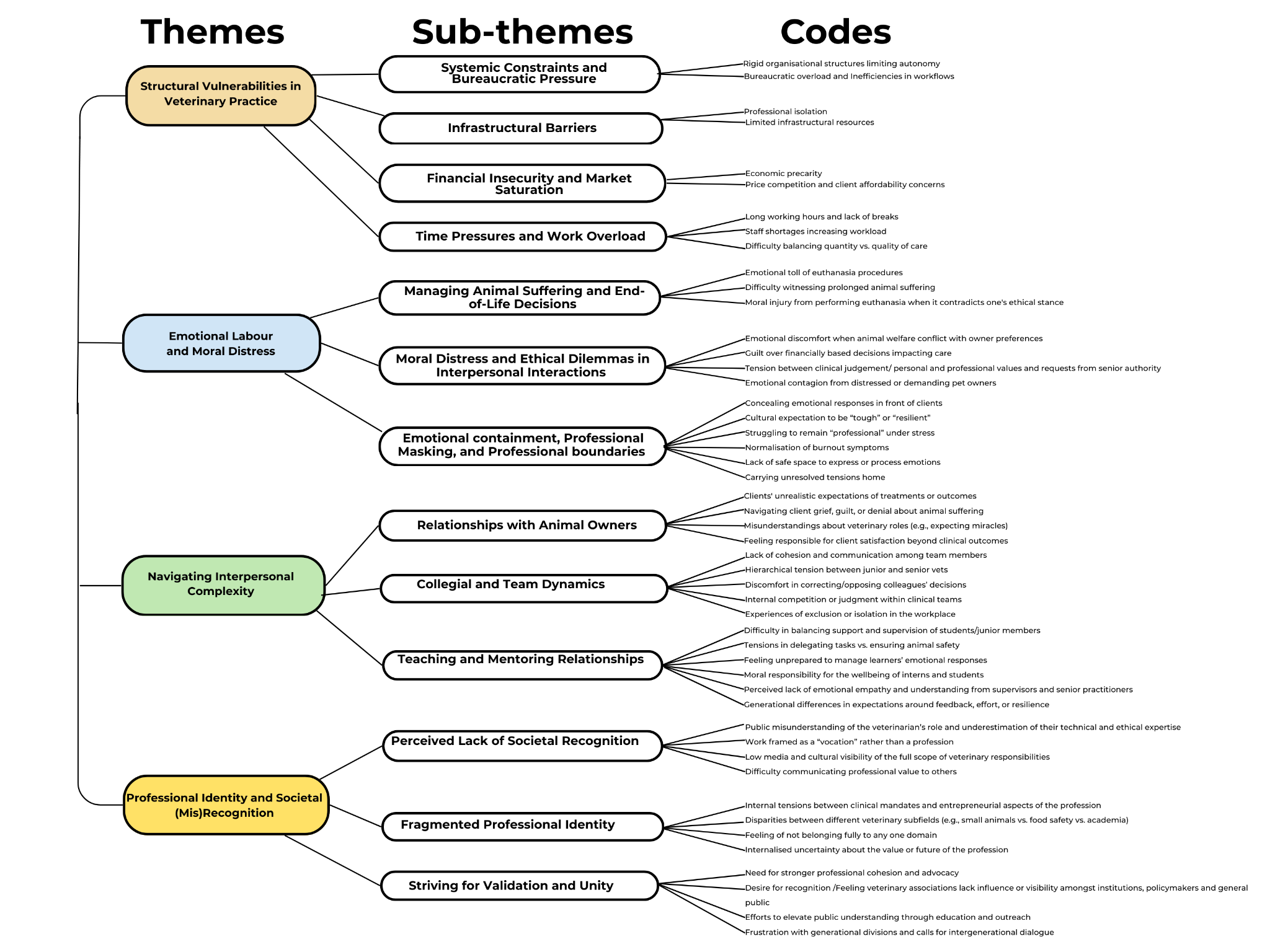

Supplement: Supplementary file 2 — Supporting File 2: vro270036‐sup‐0002‐SuppMat.docx [file VRO2-13-e70036-s003.docx]

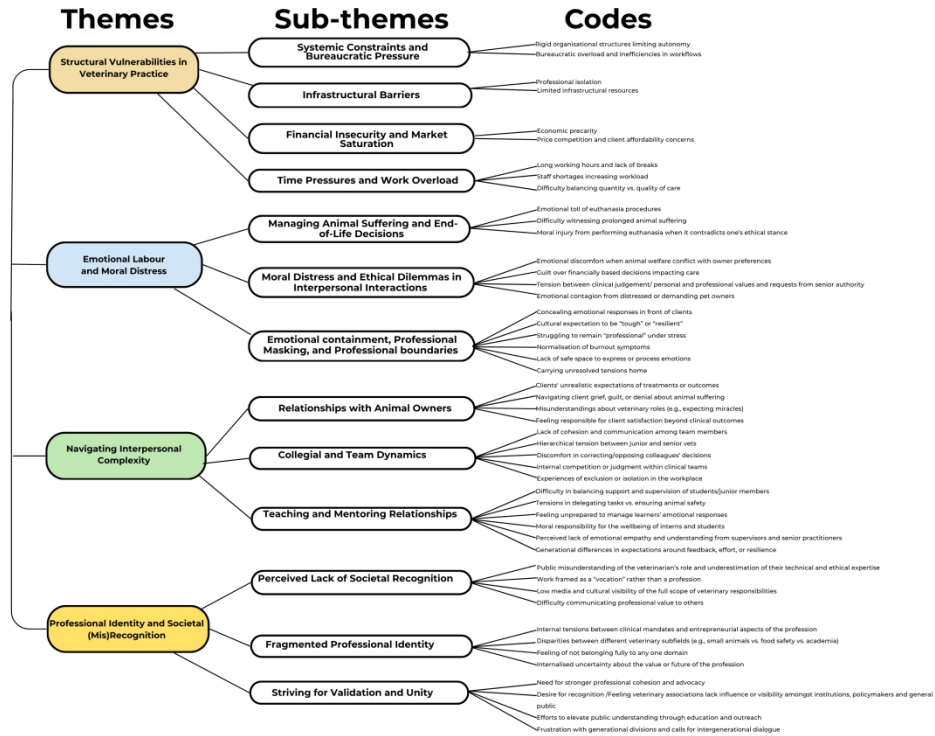

688x516mm (118 x 118 DPI)

Supplement: Supplementary file 3 — Supporting File 3: vro270036‐sup‐0003‐SuppMat.pdf [file VRO2-13-e70036-s001.pdf]
